# Supplementary material for: Functional and mutational landscapes of BRCA1 for homology-directed repair and therapy resistance
Source: eLife. 2017 Apr 11;6:e21350. doi: 10.7554/eLife.21350 (PMC5432210; doi:10.7554/eLife.21350)
Supplement: Supplementary file 1. — DOI: http://dx.doi.org/10.7554/eLife.21350.017 [file elife-21350-supp1.docx]

Supplementary file 1

Sequences of siRNAs used in this study.

NSC1 UUCGAACGUGUCACGUCAAdTdT

BRCA1-328 GGAACCUGUCUCCACAAAGdTdT

BRCA1-6284 GGAUCGAUUAUGUGACUUAdTdT

BRCA2-1949 GAAGAAUGCAGGUUUAAUAdTdT

BRCA2-2618 GCUCAAAGGUAACAAUUAUdTdT

BRCA2-4915 GGGCAAAGACCCUAAAGUAdTdT

BRCA2-9025 GGCAAAUGUUGAAUGAUAAdTdT

BRCA2-9234 GGAAAGAGAUACAGAAUUUdTdT

BRCA2-11170 CCUUAAGUCAGCAUGAUUAdTdT

PALB2-1493 UCAUUUGGAUGUCAAGAAAdTdT

PALB2-2693 GCAUAAACAUUCCGUCGAAdTdT

RAD52-1972 GGAAGGAAAUAAUCAUCUAdTdT

RAD52-2569 UCUCAGAGCAUUUCAAUUAdTdT
